# Factors Associated with the Prevalence of Hepatitis B among Volunteer Blood Donors at Jimma Blood Bank, South Ethiopia

**DOI:** 10.1155/2022/7458747

**Published:** 2022-05-23

**Authors:** Hamba Yigezu, Juhar Temam, Mitiku Bajiro, Leta Tesfaye Jule, N. Nagaprasad, Arpita Roy, Abel Saka, Krishnaraj Ramaswamy

**Affiliations:** ^1^Department of Public Health, College of Medicine and Health Science, Dambi Dollo University, Dembidolo, Ethiopia; ^2^School of Medical Laboratory, Institute of Health Science, Jimma University, Jimma, Ethiopia; ^3^Department of Medical Parasitology, Public Health Department, Dambi Dollo University, Dembidolo, Ethiopia; ^4^Department of Physics, College of Natural and Computational Science, Dambi Dollo University, Dembidolo, Ethiopia; ^5^Centre for Excellence-Indigenous Knowledge, Innovative Technology Transfer and Entrepreneurship, Dambi Dollo University, Dembidolo, Ethiopia; ^6^Department of Mechanical Engineering, ULTRA College of Engineering and Technology, Madurai 625104, Tamilnadu, India; ^7^Department of Biotechnology, School of Engineering & Technology, Sharda University, Greater Noida 201310, India; ^8^Department of Mechanical Engineering, College of Engineering and Technology, Dambi Dollo University, Dembidolo, Ethiopia

## Abstract

**Background:**

Hepatitis B is a severe, widespread infectious disease of the liver that affects millions of people around the world. It is one of the life-threatening liver infections caused by the hepatitis B virus (HBV). HBV is the cause of up to 80% of cases of primary liver cancer. Due to the potential risk associated with HBV infection, it is important to study the factors which are associated with the seropositive volunteers.

**Objective:**

The purpose of this study was to identify factors associated with seropositivity for the hepatitis B virus among volunteers who donated blood at the Jimma Blood Bank in southern Ethiopia.

**Methods:**

Cross-sectional research was conducted on blood donors who came to the Jimma Blood Bank to donate their blood. Three hundred and fifty-nine volunteer blood donors who arrived at the Jimma Blood Bank were investigated face-to-face in order to collect sociodemographic characteristics and risk factors for HBV infection. The data were analyzed using statistical software SPSS version 20.0. The association between the risk factor for HBV infection and HBV infection was determined using chi-square tests.

**Result:**

In total, there were 359 participants; their mean age was 22.5, among which 161 (44.8%) were males. Out of 359 volunteers, 13 (3.6%) were seropositive for HBsAg. The test positivity rate among males was 7/198 (3.54%), while the rate among females was 6/161 (3.7%). More than 3/4 of those who tested positive were under the age of 40. Chi-square analysis showed that volunteers whose income was between 12 and 26.84 USD were less likely to have the infectious disease than those whose income was less than 11.84 USD per month (*p*=0.042).

**Conclusion:**

The prevalence of HBV was found to be 3.6% among selected volunteers. It was found that, out of 20 volunteers, 13 had infection. Chi-square analysis showed that HBV infection was associated with low monthly income and the use of unsafe therapeutic injections.

## 1. Introduction

HBV is a well-known hepadnavirus with a double-stranded circular DNA genome [1]. A total of ten HBV genotypes (HBV-A through J) have been identified, with a wide geographic distribution, and their entire genomic sequences vary by an average of over 8%, which is important [2]. Each year, millions of people are affected by viral hepatitis, which causes disability and death in a number of countries around the world. HBV and HCV are chronically diseased in approximately 500,000,000 people worldwide [1]. The causes of viral hepatitis include primarily liver disease, which includes liver cancer, and more than 200,000 people die each year (about 2.7% of all deaths) from these causes [3]. According to the current research, HBV and/or HCV infection is responsible for around 57% of liver cirrhosis and 78% of main liver cancer [4].

According to the estimation of the World Health Organization (WHO), unsafe blood transfusions due to infection with the hepatitis B virus were 8–16 million [2]. Around 45,000 hepatitis B virus or hepatitis C virus illnesses are predicted to spread by infected transfusions in Sub-Saharan Africa each year, according to the World Health Organization [5]. Multiple studies in Ethiopia have found that the prevalence of HBV and HCV in blood donors ranges between 2.1% and 25.9% and 0.2% and 13.3%, respectively, according to the findings of [6–9]. As for the research area, there has been no previous research on blood donors in the area under consideration. Thousands of people are affected with viral hepatitis, and millions are at threat of contracting the disease. This is due to the fact that the vast majority of people who have been affected by HBV or HCV for a long period of time are completely unaware of their chronic illness. Their chances of developing the acute or chronic liver disease are high, and they run the risk of spreading the virus to others [1] through blood transfusion, or unsafe injection practices, as well as transmission from mother to baby and delivery [10]. With these concerns, the prevalence of transfusion-transmissible highly contagious diseases in blood donors is an important indicator of donor screening as well as blood safety [11]. Studies in Ethiopia looked at HBV and HCV levels in different groups of blood donors (2.1%–25% and 0.2%–13.3%) [12–14]. However, neither of these studies nor those based on three donor categories from the most recent Jimma Blood Bank were included (volunteer, replacement, and commercial). Consequently, the objective of this research is to discover whether the hepatitis B virus is prevalent in blood donors and whether there are any risk factors associated with it.

## 2. Methods

### 2.1. Study Area and Period

The study was conducted in the Jimma Blood Bank, Jimma town, located 350 km away from the country's capital city. The Jimma Blood Bank was established in 1979 in Ethiopia under the Ethiopia Red Cross Association and transferred to the Oromia Regional Health Bureau in 2004 in Ethiopia. Currently, the Jimma Blood Bank serves a population of more than 5 million and has eight catchment hospitals. Six hospitals in Oromia Jimma zone and two hospitals in Southern Nations, Nationalities, and Peoples' Region were selected for the study. The town is located at 1763 m above sea level, and its climate condition is Woyenadega, with an annual rainfall range from 1138 to 1690 mm. The Jimma town has a total population of 120,960 (CSA 2021) and has a temperature range of 11.5–27°C. The study period was conducted from Feb 1 to 30, 2021, at the Jimma Blood Bank.

### 2.2. Study Design and Population

Jimma Blood Bank conducted a cross-sectional study of volunteer blood donors to determine their preferences. Most volunteer blood donors who met donor selection criteria at the Jimma Blood Bank were included during the study period.

### 2.3. Sample Size and Sampling Technique

It was estimated that the seropositivity of important blood-borne infection between many blood donors at Bahir Dar Felege Hiwot Tertiary Hospital in Ethiopia of hepatitis B was (4.5%) with a precession of 2.25%, so the sample size was calculated using this estimate [15].

### 2.4. Data Collection

A brief explanation of the study objectives was given to the participants. Participants were asked to give their informed consent before any information was collected about them. Structured questionnaires adopted from the related literature were used to address the associated behavioural variables and the sociodemographic character.

### 2.5. Specimen Collection and Processing

From each participant, 5 ml of blood was collected using sterile capped tubes, centrifuged, the plasma was separated, and stored at 2–8°C until testing. Each plasma sample was tested for HBsAg using ELISA kits. A Human HBsAg 3rd Generation ELISA kit was used to analyze the sample.

## 3. Results

### 3.1. Baseline Characteristics of Participants

In this study, three hundred and fifty-nine (359 (100%)) volunteer blood donors were investigated. Among these, 198 (55.2%) and 161 (44.8%) were men and women, respectively. The mean age was 22.5 years, 87.2% of them were unmarried, 89.4% lived in urban areas, and 89% had a degree and above in terms of their educational level. Almost all 98.3% of the study subjects had no history of transfusion problems, as shown in Table 1. From February to August 2021, health issues for HBV infection will be assessed in the Jimma Blood Bank's volunteer blood donors.

### 3.2. Prevalence of HBV in Association with Risk Factors

When analyzed with a chi-square test, two variables were identified as statistically significant; the first one is monthly income; those earnings of 581–1,300 ETB per month were less infected with hepatitis B virus than those earning less than 580 per month ETB (*p* ≤ 0.042). Among the study subjects who had been exposed to potentially hazardous therapeutic drug injection (10 (2.8%)), 3 (30%) of them were positive for hepatitis B virus infection; therefore, compared to individuals who did not receive an injection of unsafe therapeutic medication, those who received an injection of hazardous therapeutic medication were more prone to contract an infection *p* < 0.001 (*p* < 0.001). Test positivity was high in females (3.7%), and it was higher when compared with males (3.54%), but it has no statistical significance *p* ≤ 0.923 (*p* ≤ 0.923). More than 3/4^th^ (10 (76.9%)) of the participants who tested positive were lower than 40 in terms of their age. The rate of test positivity was slightly greater among those who were not married. The rate of test positivity was slightly greater among those who were not married (2.8%) when compared to married study participants (0.8%), but there are no statistical significances *p* ≤ 0.503 (*p* ≤ 0.503).

In a similar vein, when taking into account the marital status of attendees who examined positive for HBV, trial positivity was marginally greater between unmarried attendees than between married participants (2.8% versus 0.8%), but this difference was not statistically meaningful (*p* ≤ 0.225). In spite of the fact that test positivity was comparable amongst rural and urban dwellers, 84.6% of those who screened positive were from urban regions. The positivity of the test among rural dwellers was 2 (0.6%) and 11 (11%) for those who tested positive in urban areas (3.1%) (*p* ≤ 0.636). Educated (*p* < 0.644), employed (*p* < 0.486), knew about transmission (*p* < 0.195), history of transfusion (*p* < 0.085), shared sharp materials (*p* < 0.981), exposure to surgical operations (*p* ≤ 0.247), had unprotected sex (*p* < 0.178), tattoo and ear piercing (*p* < 0.068), and tooth extraction (*p* < 0.411) were also not statistically significant, and this could be due to the homogeneity of the study sample, which consisted primarily of students who were about to graduate from the university.

When comparing the frequency of blood donation first, fewer donors test positive for HBV infection than multiple donors (2.8% vs. 0.8%) and (*p* < 0.047). According to the results of the multidimensional evaluation, individuals being paid between 581 and 26.55 USD each month were 32.2% less likely to be sick than those making less than 11.84 USD for every month. When positive, it was compared amongst other survey respondents who were classified according to average annual income (*p* < 0.042). The number of contributors who were subjected to hazardous therapeutic drug injection was 10 (2.8%), and of those, 3 (30%) tested positive for hepatitis B virus. Whether compared to volunteers who had not been exposed to the 10 (2.8%), exposed donors had a higher chance of contracting HBV than those who were not (*p* ≤ 0.001). The prevalence of HBV (13 (3.6%)) was positive for HBV.

## 4. Discussion

This study's outcomes on the prevalence of HBsAg are consistent with those reported for other Saudi Arabian regions: 3.0% in the Saudi area of Tabuk [16], 3.8% in Jazan [17], and 3.02% in Hail [18]. While higher than that of neighbouring Arab countries, including Lebanon (0.9%), Egypt (1.18%), and Oman (2.8%), it was still lower than that of the United States [16, 19, 20]. The findings of Viet et al. [21] revealed that 6.55% of healthy donors in Iran have been anti-HBc(+), with 12.2% of these donors who were also positive for HBV DNA (+). The seroprevalence rates for anti-HBc and anti-HBsAb in Iran were 4.9% and 31.9%, respectively, ten years later, according to Huang et al. [22].

According to the results of a systematic review and meta-analysis conducted worldwide, the overall seroprevalence of existing hepatitis B infections (HBsAg) among many volunteer blood donors was 2.3% (95% confidence interval). While especially in comparison with the above study, the total incidence of HBV among many volunteer contributors at the Jimma Blood Bank was 3.6%; however, when compared to this study, the prevalence of HBV was more likely to be higher in the latter. The higher HBV reported in a previous study in Bahir Dar was 4.11% [23], the higher HBV reported in Northwest Ethiopia was 25% [17], and the higher HBV reported in the regional state of Amhara and Tigray was 6.2% [16]. This could be caused by the fact that the blood bank only accepts voluntary donors, with no consideration given to replacement or commercial donors. Compared to volunteer donors, all replacement donors (53.6%) and commercial donors (56.6%) have a higher percentage of donors [17]. When the findings of this study were compared with similar studies in other countries, a higher percentage was reported from southern Darfur (6.25%) [24], Tete in Mozambique (10.6%) [25], Kano in Nigeria (11.1%) [26], Ibadan in Nigeria (5.9%) [27], Akura in Nigeria (7.4%) [28], Quang Tri in Vietnam (11.1%) [29], and Aden city in Yemen (5.1%) [30].

Lower finding was also reported from Kathmandu in Nepal (0.47%) [31], Babylon in Iraq (0.7%) [19], Jordan (1.4%) [20], and Gujarat in India (0.68%) [25]. Specific geographic locations, sociocultural differences, dominant genotypes, subgenes, and mutant existence are all possible factors to take into consideration, among others.

In this study, a statistically significant relationship was discovered between the income status and HBV. If the study participants were divided into groups based on their average monthly income, those earnings of 12–26.55 USD were 32.2% less likely to be infected than those earning less than 11.84 USD per month (*p*=0.042). This could imply that, as people's socioeconomic status improves, they are more likely to take precautions against HBV-related factors. This was similar to but higher by percentage, than the findings in Jimma, Southwest Ethiopia, where the seroprevalence of HBsAg, as well as its risk factors among many pregnant women with incomes less than 10.21 USD/month, was 88.9% (p0.05), but lower by percentage than the findings in Jimma despite the fact that the rapid chromatographic immunoassay test was used in those studies [32]. In addition, the use of unsafe therapeutic injection (*p*=0.000) was found to be associated with a higher risk of miscarriage. This finding was consistent with a study conducted among many pregnant women in the city of Bahir Dar, in North West Ethiopia, which reported an important association with unsafe therapeutic injection in 15.9% of cases (AOR = 5.65, 95% confidence interval: 1.44–22.19) [33]. An additional consensus was reached on the fact that, in evolving countries, exposures to infected therapeutic injection facilities are common in a variety of settings. In 2000, contaminated injections were responsible for approximately 21 million HBV infections worldwide, accounting for 32% of all new HBV infections [1].

## 5. Conclusion

According to the findings of this study, the prevalence of HBV among voluntary blood donors at the Jimma Blood Bank was 3.6%. HBV was detected in 13 of the 20 participants. Males constituted 3.5% of the total detection. The test positivity rate among males was 7/198 (3.54%), while the rate among females was 6/161 (3.7%). More than 3/4 of those who tested positive were under the age of 40. When the results of the chi-square analysis were tried to compare among many study participants who were divided into groups based on their average household salary, participants' earnings 12–26.84 USD were less likely to have the infectious disease as compared to those earning less than 11.84 USD per month (*p*=0.042). Volunteer donors were revealed to dangerous therapeutic drug injection in 10 cases (2.8%), and of those, 3 (30%) tested positive for hepatitis B virus (HBV). Donors who had been exposed had a higher risk of contracting HBV specially than those volunteer donors who had not been exposed (*p*=0.000)

### 5.1. Recommendation

Improved community awareness, infection control, and posttreatment counselling will all be implemented to keep infections under control.

## Figures and Tables

**Table 1:** Sociodemographic characteristics of participants in association with the HBV.

Variable		Total N (%)	HBsAg		*p* value (*x*^*2*^ test)
			Negative N (%)	Positive N (%)	
Sex	Male	198 (55.2)	191 (53.2)	7 (1.9)	0.923
	Female	161 (44.8)	155 (43.2)	6 (1.7)	
Age	18–30	152 (42.3)	145 (40.4)	7 (1.9)	0.503
	31–40	137 (38.2)	134 (37.3)	3 (0.8)	
	41–65	70 (19.5)	67 (18.7)	3 (0.8)	
Occupation	Student	277 (77.2)	268 (74.7)	9 (2.5)	0.486
	Employed	65 (18.1)	62 (17.3)	3 (0.8)	
	Merchant	7 (1.9)	7 (1.9)	0 (0.0)	
	Others	10 (2.8)	9 (2.5)	1 (0.3)	
Educational status	9–12	14 (3.9)	13 (3.6)	1 (0.3)	0.644
	TVET diploma	27 (7.5)	25 (7.0)	2 (0.6)	
	Degree and above	318 (88.6)	308 (85.8)	10 (2.8)	
Marital status	Unmarried	313 (87.2)	303 (84.4)	10 (2.8)	0.225
	Married	46 (12.8)	43 (12.0)	3 (0.8)	
Residence	Urban	321 (89.4)	310 (86.4)	11 (3.1)	0.636
	Rural	38 (10.6)	36 (10.0)	2 (0.6)	
Income(ETB)	<580	90 (25.1)	82 (22.8)	8 (2.2)	0.042
	581–1300	204 (56.8)	44 (12.6)	4 (1.1)	
	1301–4000	46 (12.8)	45 (12.5)	1 (0.3)	
	4001–8500	19 (5.3)	19 (5.3)	0 (0.0)	
Know about transmission	Yes	145 (40.4)	142 (39.40	3 (0.8)	0.195
	No	214 (59.6)	204 (56.8)	10 (2.8)	
Donor status	First	253 (70.3)	143 (39.8)	10 (2.8)	0.047
	Multiple	106 (29.5)	103 (28.6)	3 (0.8)	
History of transfusion	Yes	6 (1.7)	5 (1.4)	1 (0.3)	0.085
	No	353 (98.3)	341 (94.9)	12 (3.3)	
Unsafe therapeutic drug injection	Yes	10 (2.8)	7 (1.9)	3 (0.8)	0.001
	No	349 (97.2)	339 (94.4)	10 (2.8)	
Share razor and sharp materials	Yes	27 (7.5)	26 (7.2)	1 (0.3)	0.981
	No	332 (92.5)	320 (89.1)	12 (3.3)	
Surgical procedure	Yes	107 (29.8)	105 (29.2)	2 (0.6)	0.247
	No	252 (70.2)	241 (67.1)	11 (3.1)	
Unsafe multiple sexual activity	Yes	117 (32.6)	115 (32.0)	2 (0.6)	0.178
	No	242 (67.4)	231 (64.3)	11 (3.1)	
Tattoo, body, or ear piecing	Yes	172 (47.9)	169 (47.0)	3 (0.8)	0.068
	No	187 (52.1)	177 (49.3)	10 (2.8)	
Tooth extraction	Yes	57 (15.9)	56 (15.5)	1 (0.3)	0.411
	No	302 (84.1)	290 (80.7)	12 (3.3)	

## Data Availability

The data used to support the findings of this study are included within the article.
